# Supplementary material for: The interactome of intact mitochondria by cross-linking mass spectrometry provides evidence for coexisting respiratory supercomplexes
Source: Mol Cell Proteomics. 2017 Dec 8;17(2):216–32. doi: 10.1074/mcp.RA117.000470 (PMC5795388; doi:10.1074/mcp.RA117.000470)
Supplement: Supplemental Data [file supp_RA117.000470_133922_0_supp_23978_fzffwf.zip › spectra_annotation/mito_spectra_annotation/3-1-3-1-1-1.pdf]

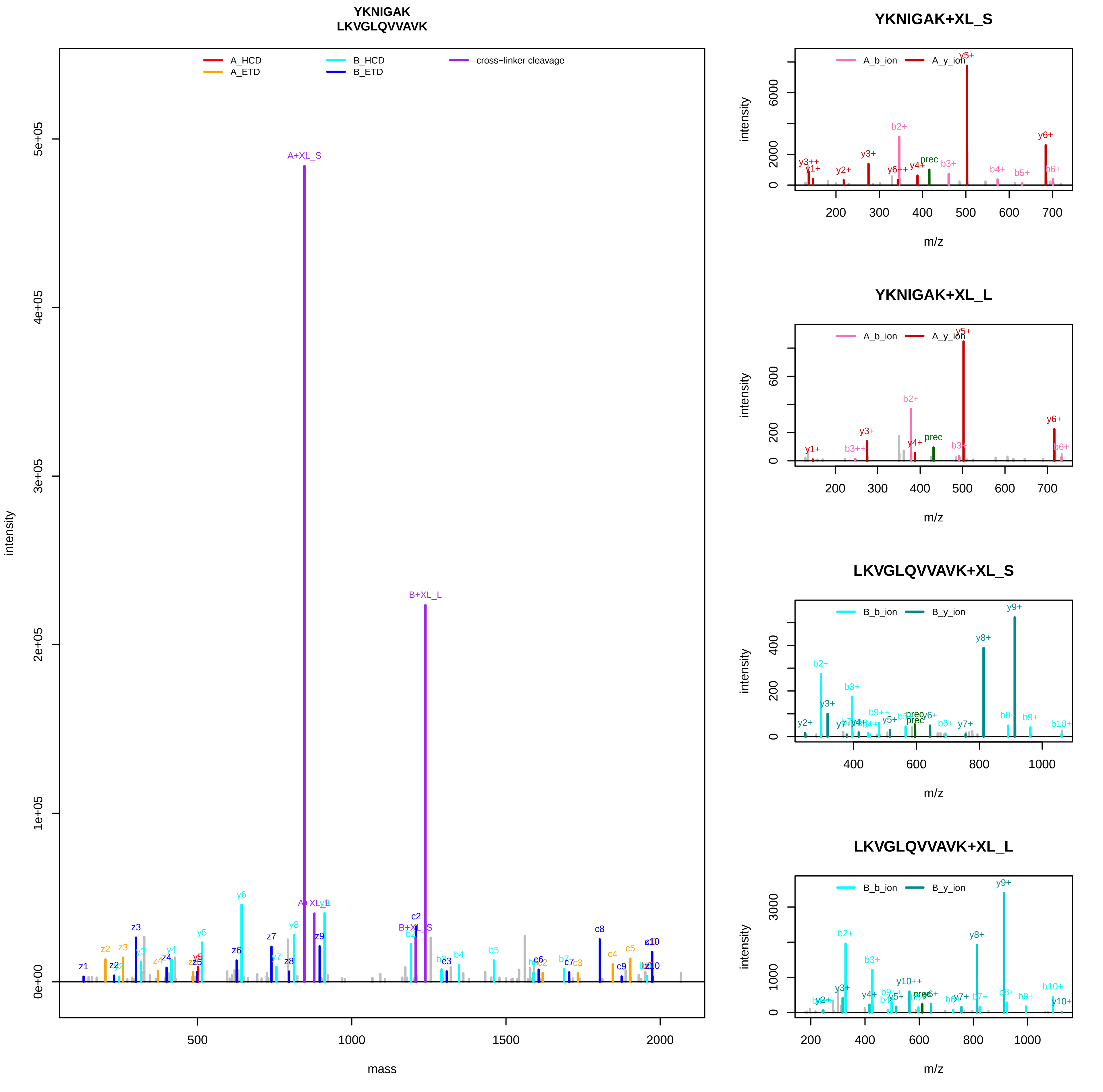

YKNIGAK
LKVGLQVVAVK
A_HCD
A_ETD
B_HCD
B_ETD
cross−linker cleavage
intensity
mass
0e+00
1e+05
2e+05
3e+05
4e+05
5e+05
500
1000
1500
2000
A+XL_S
B+XL_L
y6
y5
y8
z3
z7
c2
c8
z10
YKNIGAK+XL_S
A_b_ion
A_y_ion
y5+
b2+
y6+
y3+
prec
m/z
YKNIGAK+XL_L
LKVGLQVVAVK+XL_S
B_b_ion
B_y_ion
y9+
y8+
b2+
b3+
LKVGLQVVAVK+XL_L
